# Therapeutic plasma exchange in adolescent and adult patients with autoimmune neuropsychiatric disorders associated with streptococcal infections

**DOI:** 10.1002/jca.22023

**Published:** 2022-10-17

**Authors:** Kristina Prus, Krystol Weidner, Caroline Alquist

**Affiliations:** ^1^ Biomedical Services American Red Cross Portland Oregon USA; ^2^ Hoxworth Blood Center University of Cincinnati Cincinnati Ohio USA

## Abstract

Pediatric autoimmune neuropsychiatric disorders associated with streptococcal (PANDAS) infections and pediatric acute‐onset neuropsychiatric syndrome (PANS) are typically diagnosed in childhood. Therapeutic plasma exchange (TPE) has been recommended to remove relevant antibodies and treat symptomatic presentations in children and adolescents, but there are no studies that evaluate the use of TPE in patients who are diagnosed later in life. It is therefore unclear if using an accepted treatment for pediatric PANS/PANDAS patients would be beneficial in adults with prolonged PANDAS/PANS symptomatic histories. This study investigated 16 late adolescent and adult PANDAS/PANS patients' responses to TPE. Improvement was noted in over half of the patients with available follow‐up information.

## 1 BRIEF REPORT

Pediatric autoimmune neuropsychiatric disorders associated with streptococcal (PANDAS) infections and pediatric acute‐onset neuropsychiatric syndromes (PANS) are post‐infectious autoimmune neuropsychiatric disorders associated with Group‐A beta‐hemolytic streptococcus infection. PANDAS/PANS are typically diagnosed in childhood and are characterized by a sudden development of new disordered behaviors including obsessions and compulsions.^1^, ^2^ Limited and conflicting studies exist regarding PANDAS/PANS, including very few studies on biomarkers, diagnosis, and treatment.^1^, ^2^ According to the 2019 American Society for Apheresis guidelines, the use of therapeutic plasma exchange (TPE) in PANDAS patients with an exacerbation carries a category II indication, denoting it as a second line therapy.^3^ As studies have shown a positive correlation between anti‐neuronal antibodies and PANDAS/PANS diagnosis, TPE has been implemented to remove these antibodies and treat symptomatic presentations.^2^, ^4^, ^5^ One study showed a benefit of TPE in pediatric patients with an average of 4.2‐years of symptomatic disease course prior to initiation of TPE.^5^ However, there are no studies that evaluate the use of TPE in patients who are diagnosed with PANDAS/PANS later in life, despite pediatric or peri‐pubertal age of onset. It is therefore unclear if using an accepted treatment for pediatric PANS/PANDAS patients would be beneficial in adults with prolonged PANDAS/PANS symptomatic histories. This study aimed to investigate late adolescent and adult PANDAS/PANS patients' responses to TPE.

We conducted an Institutional Review Board‐approved retrospective chart review on all patients treated with TPE for PANDAS/PANS indications via a single outpatient apheresis service located in a medium‐sized urban medical center. Each patient was managed by an outside local physician. Available documents included clinic notes and laboratory results provided to the apheresis service by the referring physician, electronic medical records, and TPE procedural records. Each record was independently reviewed by two investigators for accuracy. Medical records including laboratory results and notes were reviewed and patient age, gender, diagnoses, TPE procedural details, medication and treatment history, pre‐treatment antineuronal antibody evaluation (Cunningham Panel), pre‐treatment anti‐streptolysin (ASO) titers, and response to treatment modalities were recorded, as available.

In total, 16 patients were identified (aged 14–41 years, median 19.5 years). Four patients were under 18 years old, and half were 20 years or older. All patients had a reported race of Caucasian. Five (31%) of the patients were male. All patients had a diagnosis of PANDAS or PANS. Age at diagnosis ranged from 11–22 years old, with a median of 16 years. We were unable to discern dates of symptom onset from available data. Eight patients had recorded concurrent psychiatric comorbidities, including obsessive‐compulsive disorder (OCD) (n = 6), anxiety (n = 3), autism, bipolar disorder, depression, and schizophrenia (n = 1). Five of these patients had at least two different diagnoses. Twelve patient records revealed previous medication‐based psychiatric treatments prior to TPE referral. Eleven of those patients remained on psychiatric medications at the time of TPE initiation.

Anti‐neuronal antibodies to dopamine receptor D1, dopamine receptor D2L, lysoganglioside GM1, and tubulin anti‐antibodies, in addition to the production of calcium/calmodulin‐dependent protein kinase II in response to these autoantibodies, can be assessed together via a Cunningham Panel. Twelve patient records included Cunningham Panel results (75% of cohort), which are summarized in parallel with available gender (100%), anti‐streptolysin titers (43%), and documented response (38%) data in Table 1.

**TABLE 1:** Sixteen patients listed by gender, age, pre‐treatment Cunningham Panel findings, pre‐treatment peak ASO titers, TPE treatment days/number of courses, and documented improvement. Color key of Cunningham Panel: Elevated levels (orange); Borderline levels (yellow) and normal values (blue).

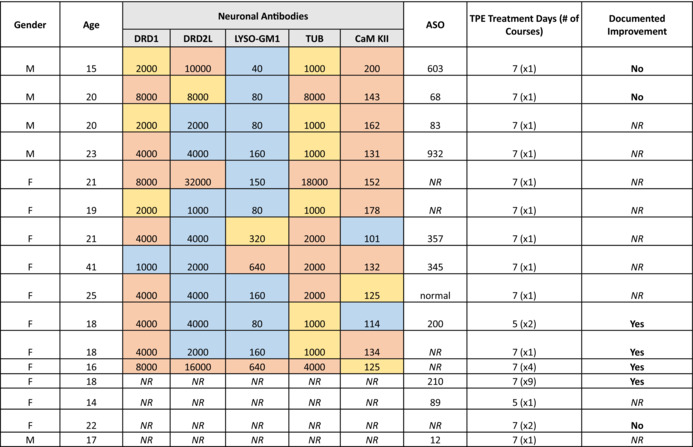

Abbreviations: ASO, anti‐streptolysin titer; CaM KII, CaM Kinase II (% of baseline); DRD1, anti‐dopamine receptor D1 (titer); DRD2L, anti‐dopamine receptor D2L (titer); F, female; LYSO‐GM1, anti‐lysoganglioside GM1 (titer); NR, not reported; TUB, anti‐tubulin (titer).

Prior to TPE, most patients had received other PANDAS/PANS‐oriented treatments. Seven patients had completed at least one course of antibiotics, usually with azithromycin though two patients used amoxicillin‐clavulanic acid. Two patients reported use of steroids.

For TPE treatment, one course was defined as five or seven single plasma volume TPE with 5% albumin replacement and citrate anticoagulant, utilizing a Spectra Optia. Fourteen (5 male, 9 female) received 7 days of treatment, while the remaining two female patients received 5 days of TPE treatment, all scheduled every other day (excluding weekends). Four female patients were treated with multiple courses of TPE, receiving 1, 2, 3, or 8 additional courses. The longest duration of treatment was over nine courses, performed for symptomatic indications. Ages of those receiving multiple courses were 16, 18, 18, and 22 years. No adverse reactions or complications of apheresis treatment were identified.

Seven patients had recorded post‐TPE PANDAS/PANS responses, denoting improved (n = 4) or not improved (n = 3). Improvement after TPE was noted 1–10 days after treatment in four females aged 16–18. Three of these responders received subsequent TPE courses for PANDAS/PANS exacerbations. Of the non‐responders, two were male aged 15 and 20 years, and one was female aged 22 years. The female patient received two courses of TPE (each course being 5 TPE). The male patients each completed a single 7 TPE course. Cunningham Panel results did not correlate with the reported response to TPE. Both responders and non‐responders had elevated anti‐dopamine receptor D1 and anti‐tubulin levels. One non‐responder and one responder showed elevated anti‐dopamine receptor D2L levels. Elevation of all titers may predict response, but only one responder displayed this pattern.

In our cohort, no available test value appeared to predict TPE response (Table 1). Improvement was noted in over half of the patients with available follow‐up information and no adverse reactions to TPE were documented. TPE appears to be a safe and potentially efficacious treatment option for symptomatic adolescent and adult PANDAS/PANS patients. Limitations of this study include the retrospective nature and incomplete data availability, including post‐treatment ASO and Cunningham Panel results.

## Data Availability

Research data are not shared.
